# Natural Language Processing-Based Triage of Superficial Soft Tissue Ultrasound Reports in Orthopedic Practice

**DOI:** 10.3390/diagnostics16071068

**Published:** 2026-04-02

**Authors:** Nuri Koray Ülgen, Mevlüt Aytaç Demir, Ali Said Nazlıgül, Nihat Yiğit, Sadık Emre Erginoğlu, Ünal Demir, Mehmet Orçun Akkurt

**Affiliations:** 1 Orthopaedics and Traumatology Clinic, Sincan Training and Research Hospital, University of Health Sciences, Ankara 06949, Türkiye; alisaid012@gmail.com (A.S.N.); nihatyigit3@gmail.com (N.Y.); seerginoglu@gmail.com (S.E.E.); unal61demir@gmail.com (Ü.D.); mehorcun@gmail.com (M.O.A.); 2 Department of Electrical and Electronics Engineering, Faculty of Engineering Architecture and Design, Bartin University, Bartin 74110, Türkiye

**Keywords:** natural language processing, radiology reports, superficial soft tissue ultrasound, urgency classification, musculoskeletal disorders

## Abstract

**Background/Objectives**: Natural language processing (NLP) has emerged as a promising approach for extracting clinically meaningful information from unstructured radiology reports. While most artificial intelligence applications in musculoskeletal imaging focus on image-based analysis, the potential of NLP for urgency assessment in superficial soft tissue ultrasound reports remains underexplored. This study aimed to develop and evaluate an NLP-based triage model to classify superficial soft tissue ultrasound reports according to clinical urgency in orthopedic practice. **Methods**: A curated dataset of superficial soft tissue ultrasound reports requested for palpable soft tissue masses and subcutaneous swellings was retrospectively collected from routine orthopedic outpatient practice. Reports were manually annotated into three triage categories: non-pathological (GREEN), non-urgent pathological (YELLOW), and urgent or potentially urgent findings (RED). A pretrained Turkish BERT model was fine-tuned for three-class classification. Model performance was evaluated using accuracy, macro-averaged F1 score, per-class precision and recall, and confusion matrices. An independent dataset of previously unseen reports was additionally used to assess robustness under real-world conditions. **Results**: After preprocessing and deduplication, 394 unique report segments were included. The baseline BERT model achieved an accuracy of 92.5% and a macro-averaged F1 score of 0.9106 on the test set. High classification performance was observed across all classes, with particularly reliable detection of RED reports representing urgent clinical conditions. External evaluation on independent reports demonstrated high agreement with physician annotations, with discrepancies mainly occurring in borderline or indeterminate cases. **Conclusions**: This study demonstrates that NLP-based analysis of superficial soft tissue ultrasound reports can effectively support urgency assessment in orthopedic practice. The proposed approach offers a practical, scalable, and image-independent solution for triage, with potential to improve workflow efficiency and facilitate timely clinical decision-making in musculoskeletal imaging.

## 1. Introduction

Artificial intelligence (AI)-based applications have become an integral part of everyday life across a wide range of domains. In recent years, the application of AI in medicine has expanded considerably [1,2,3]. However, its routine integration into clinical practice remains limited. Most medical AI studies are based on machine learning (ML) and deep learning (DL) approaches, with particularly strong performance reported in image-based tasks such as radiologic image interpretation and automated detection systems [4,5].

Natural language processing (NLP), a core subfield of machine learning, focuses on the extraction of meaningful and analyzable information from unstructured text data. In healthcare, NLP enables the automated analysis of large volumes of free-text clinical documentation, including electronic health records, discharge summaries, and radiology reports [6]. Although the use of NLP in medical applications predates the recent surge of AI technologies in daily life, its clinical potential has gained renewed attention with the increasing availability of large-scale digital medical data [7,8]. Previous studies have demonstrated that NLP-based systems can efficiently extract clinically relevant information, significantly reduce manual data abstraction workloads, and support clinical decision-making processes [9].

Radiology reports play a central role in clinical workflows by translating imaging findings into clinically interpretable information. Although artificial intelligence has achieved substantial success in image-based analysis, the accompanying narrative radiology reports remain a relatively underexplored source of structured clinical information. Advances in natural language processing enable the systematic analysis of these free-text reports, offering the potential to extract clinically actionable insights that may not be readily apparent in routine practice [10].

In the field of orthopedics and traumatology, the majority of ML-based studies have focused on image processing techniques applied to radiographs, computed tomography, or magnetic resonance imaging [11]. In contrast, NLP-based applications in orthopedics are relatively scarce and have primarily concentrated on risk prediction models derived from clinical notes [12,13]. Studies employing NLP to analyze radiology reports have largely focused on specific areas such as breast and thyroid ultrasonography, where NLP has been used to assess lesion characteristics and estimate malignancy risk with promising results [14,15]. However, the application of NLP-based applications to musculoskeletal soft tissue masses particularly within the context of orthopedic oncology remains limited.

Although NLP-based triage and urgency classification of radiology reports have been explored in prior studies, the present work differs in several important aspects. To the best of our knowledge, this is the first study focusing specifically on superficial soft tissue ultrasound reports requested for palpable masses and subcutaneous swellings in orthopedic practice. This clinical context presents unique challenges, including short, heterogeneous, and non-templated reports that frequently contain indeterminate language and overlap between oncologic suspicion and acute non-neoplastic conditions such as hematoma or inflammatory collections. Unlike prior work that primarily addresses either oncologic risk stratification or emergency department triage, our approach integrates both malignancy-related and acute musculoskeletal urgency within a single operational triage framework. These characteristics extend the application of NLP-based triage beyond language-specific considerations and highlight its potential utility in a previously underexplored area of musculoskeletal imaging.

Previous research has suggested that NLP-based triage and urgency assessment using clinical text can improve workflow efficiency and reduce delays in patient management [16,17]. In routine orthopedic practice, superficial soft tissue masses are frequently evaluated using ultrasonography, and the interpretation of ultrasound reports plays a key role in determining the need for further imaging, referral, or urgent intervention. Delays in identifying potentially concerning findings may result in postponed diagnosis and treatment, especially in cases with malignant potential.

In this study, we aimed to develop an NLP-based machine learning model to classify superficial soft tissue ultrasound reports requested in an orthopedic outpatient setting according to clinical urgency. The proposed model categorizes ultrasound reports into three groups: those requiring urgent further evaluation, those not requiring urgent intervention, and those without pathological findings. By enabling the automated identification of reports that warrant prompt clinical attention, this approach offers a scalable and practical framework for supporting clinical triage and improving efficiency in musculoskeletal imaging workflows. The findings of this study highlight the potential role of NLP-based systems in risk stratification of radiology reports and suggest that such approaches may be extended to other domains of oncologic and musculoskeletal imaging.

## 2. Materials and Methods

### 2.1. Dataset and Clinical Context

The study was conducted in accordance with the Declaration of Helsinki and approved by the Ethics Committee of Sincan Training and Research Hospital (approval code: BAEK-2025-65; date of approval: 24 June 2025). This study was conducted on a curated dataset of superficial musculoskeletal ultrasonography (USG) reports obtained from routine orthopedic clinical practice. The reports correspond to superficial soft tissue ultrasound examinations requested for the evaluation of palpable soft tissue masses and subcutaneous swellings. All examinations were requested by orthopedic surgeons following clinical evaluation and were subsequently performed and reported by radiologists. The dataset reflects a heterogeneous reporting environment, as the reports were authored by multiple radiologists rather than a single reader, thereby capturing real-world variability in reporting style and terminology. The study period spanned from 1 January 2023 to 31 December 2024.

Typical examination indications included suspected ganglion cysts, small cystic or solid lesions, localized swelling, post-traumatic soft tissue changes (e.g., edema or hematoma), tendon-related abnormalities, and periarticular soft tissue conditions. A substantial proportion of reports explicitly documented the absence of a discrete lesion or clinically significant pathology, frequently accompanied by recommendations for clinical follow-up or additional imaging if symptoms persisted.

All reports consisted of short, free-text radiology findings dictated by physicians, reflecting real-world orthopedic ultrasound reporting practices. No imaging data were used; the study relied exclusively on textual report segments.

For the purposes of this study, the unit of analysis was defined as a report segment corresponding to the free-text “Findings” or “Conclusion” section of a superficial soft tissue ultrasound report. In routine clinical practice at our institution, such ultrasound reports are generally short and focused, often consisting of a single descriptive paragraph. Accordingly, each report typically constituted a single report segment.

In rare cases where a report contained more than one distinct descriptive section (e.g., separate comments on different anatomical regions), these sections were treated as a single combined segment to preserve the original clinical context. Each report segment originated from a single ultrasound examination. All train/validation/test splits were performed at the patient level to ensure that no patient appeared in more than one split (Section 2.4). Representative examples of report segments for each triage category are provided in Appendix A.

To minimize potential information leakage, dataset splitting was performed at the ultrasound examination level rather than at the raw text level. Because each report segment originated from a unique ultrasound examination and no report generated multiple segments, this approach effectively ensured patient- and examination-level separation between the training, validation, and test sets.

In addition, prior to dataset splitting, a structured deduplication process was applied to remove identical or near-identical report texts, thereby reducing the risk of repeated patterns or highly similar expressions appearing across different dataset splits. As a result, no identical report segments were shared between the training, validation, and test sets.

### 2.2. Annotation Protocol and Triage Labels

Each report segment was manually annotated by expert physicians into one of three clinically motivated triage categories:

- GREEN: No clinically significant musculoskeletal soft tissue abnormality detected (e.g., normal findings or explicit absence of a lesion).
- YELLOW: Non-urgent musculoskeletal findings, including benign or indeterminate lesions (e.g., ganglion cysts, small cystic formations, mild edema, or tendon changes) that may require follow-up or clinical correlation.
- RED: Clinically significant or potentially urgent musculoskeletal pathology, including findings suggestive of hematoma, partial muscle or tendon rupture, inflammatory collections, or mass-like lesions raising suspicion of malignancy, as well as other findings warranting prompt further diagnostic evaluation.

Annotations were derived from routine clinical decision-making criteria rather than research-driven labeling rules.

Reports were independently annotated by two board-certified orthopedic specialists (N.K.Ü., N.Y.) following a predefined triage guideline (GREEN/YELLOW/RED). Initial labels were assigned in parallel and were subsequently reconciled through a consensus adjudication meeting. In case of disagreement, the final label was determined by discussion of the clinical context and diagnostic descriptors until consensus was reached. Annotators were blinded to model outputs during labeling. Because the final labels reflect consensus adjudication rather than independent final decisions, formal inter-rater agreement statistics (e.g., Cohen’s κ) were not reported.

### 2.3. Data Cleaning, Segment Extraction, and Deduplication Strategy

All ultrasound reports were provided as individual PDF files, each corresponding to a single finalized superficial musculoskeletal soft tissue ultrasound examination. Text was extracted page-wise using the pdfplumber library (v 0.11.9) and subsequently normalized using Unicode NFKC normalization and whitespace standardization. A “report segment” was defined as the extracted free-text findings content from a single report (one PDF corresponds to one examination report).

Segment extraction followed a deterministic, rule-based pipeline. We first located the main heading “TETKİK SONUCU”, then identified the examination sub-header matching “Yüzeyel/Yüzeysel Doku” together with modality variants (US/U/S/USG/Ultrason/Ultrasonografi). Pattern matching was performed on a simplified search string, and match boundaries were projected back onto the original extracted text using a character-level simplified-to-raw index map to prevent index drift. The segment began immediately after the sub-header and ended at the earliest occurrence of (i) a separator line, (ii) a technical note line (e.g., “Ultrason cihazından kaynaklanan…”), or (iii) a clinician signature line (e.g., “Uzm. Dr.”, “Prof. Dr.”).

Each report yielded at most one segment (or none if required anchors were absent). Across 398 reports, 397 segments were initially extracted. After cleaning and deduplication, 396 unique segments remained. Two records lacked clinician-provided labels and were excluded from supervised modeling, yielding a final labeled dataset of 394 segments (Table 1). Duplicate segments were identified based on identical normalized text. When identical report texts were associated with conflicting labels, we retained the YELLOW label as a conservative triage decision and performed a sensitivity analysis in which conflicted duplicates were removed; results were comparable and are reported in Section 3.5 and Appendix A. Three anonymized representative examples (GREEN/YELLOW/RED) are provided in Appendix A to illustrate typical model inputs.

### 2.4. Train–Validation–Test Split and Leakage Control

We evaluated models under two complementary splitting strategies to assess potential leakage from repeated templates and to quantify robustness. First, we used a standard stratified split (SEED = 42) to preserve class proportions. Second, we performed a patient-level split to ensure that reports originating from the same patient never appeared in both training and test sets.

For patient-level splitting, we derived a patient identifier (patient ID) from the report filename by extracting the leading numeric token, and applied GroupShuffleSplit to allocate patients into training (80%), validation (10%), and test (10%) partitions. We verified group disjointness across partitions to prevent leakage. For both split strategies, the resulting sample counts were: training *n* = 315, validation *n* = 39, and test *n* = 40.

### 2.5. Independent Evaluation Dataset

To assess model robustness under real-world conditions, an independent evaluation dataset was constructed. This dataset consisted of 39 superficial soft tissue ultrasound reports obtained in 2022 that were not included in the training, validation, or test sets and had not been previously reviewed or labeled during model development. These reports were collected outside the model training pipeline to avoid information leakage and to simulate real-world deployment conditions. Model predictions on this independent dataset were compared against annotations provided by physicians who were not involved in the initial labeling process.

### 2.6. Baseline Language Model (Model A: Base BERT)

We fine-tuned a pretrained Turkish BERT model (dbmdz/bert-base-turkish-cased) for 3-way sequence classification. Text was truncated to MAX_LEN = 256 tokens. Training used AdamW with learning rate 2 × 10^−5^ and weight decay 0.01, and was performed for a fixed maximum of 6 epochs, selecting the best checkpoint by validation macro-F1. Evaluation metrics were accuracy, macro-F1, and weighted-F1.

Formally, given a tokenized report segment $x=(x_{1},\ldots ,x_{T})$, the encoder produces a pooled representation $h=h_{\left[CLS\right]}$. Classification logits are computed as z=Wh+b, class probabilities as p=softmax(z), and the predicted triage label as $\hat{y}=arg\;{max}_{c}\;p_{c}$.

### 2.7. Keyword Fusion Model (Model B: BERT + Keyword Fusion)

We investigated a late-fusion approach combining neural logits with a keyword-derived score vector. A clinician-provided triage dictionary (GREEN/YELLOW/RED phrase sets) was compiled into normalized phrase lists; phrase overlaps were resolved with priority RED > YELLOW > GREEN to reduce contradictory matching. For each text, a 3D keyword count vector K was computed (hit counts per class). Fusion was performed as follows.

Let $K\in R^{3}$ denote the keyword hit-count vector for the three triage classes, and let z denote the BERT logits. We define the fused logits as $z_{fused}=z+\alpha K$ and compute fused probabilities as $p_{fused}=softmax(z_{fused})$; the fused prediction is ${\hat{y}}_{fused}=arg\;{max}_{c}\;p_{fused,c}$. The fusion weight α was treated as a validation-tuned hyperparameter (Section 3.7). Because tuning did not yield clinically meaningful improvements, the keyword-fusion component is not included in the final system and is reported as an exploratory analysis.

### 2.8. Regex Rule System (Models C and D)

We also evaluated a lightweight, deterministic post-processing layer based on regular expressions designed to reduce clinically implausible outputs:

- Negative shield: patterns indicating explicitly negative examinations (e.g., “kitle izlenmedi”, “lezyon saptanmadı”) intended to prevent inappropriate escalation to RED.
- Red force: patterns indicating high-risk concepts or explicit escalation language (e.g., “hematom”, “apse”, “MRG önerilir” in specific contexts, suspicious descriptors) intended to up-triage when the model under-calls.

Model variants:

- Model C: regex applied on top of Model A predictions.
- Model D: regex applied on top of Model B fusion predictions.

We report the number of predictions changed by regex as an operational measure of rule intervention intensity.

Formally, let $r_{neg}(x)$ and $r_{red}(x)$ be indicator functions such that $r_{neg}(x)=1$ if the segment x matches any negative-shield pattern and $r_{red}(x)=1$ if x matches any red-force pattern (otherwise the indicators are 0). Given a base prediction $\hat{y}$, the final decision ${\hat{y}}^{*}$ is defined by the following override rules: if $r_{neg}(x)=1$, then ${\hat{y}}^{*}=GREEN$; else if $r_{red}(x)=1$, then ${\hat{y}}^{*}=RED$; otherwise ${\hat{y}}^{*}=\hat{y}$. If both indicators are triggered, the negative-shield rule takes precedence to prevent unsafe escalation in explicitly negative reports.

### 2.9. Comparative Evaluation Protocol

Due to the limited size of the internal test set, non-parametric bootstrapping with 1000 resamples was used to estimate 95% confidence intervals for accuracy and macro-averaged F1 score for the primary model (Model A) on the held-out test set (*n* = 40). For comparative analyses based on cross-validation, we report mean performance with 95% uncertainty intervals across folds (Table 2). Performance was summarized using accuracy, macro-F1, weighted-F1, per-class F1, and confusion matrices (class order: GREEN, YELLOW, RED).

### 2.10. Training Details

Model development and evaluation were implemented in Python 3.12.12 using the transformers library (v5.1.0), PyTorch (v2.9.0), and scikit-learn (v1.6.1). All input texts were truncated to a maximum length of 256 tokens. The primary model was dbmdz/bert-base-turkish-cased; additional transformer comparisons used dbmdz/distilbert-base-turkish-cased and xlm-roberta-base.

Models were fine-tuned using the AdamW optimizer (torch.optim.AdamW; Transformers v5.1.0) (learning rate 2 × 10^−5^, weight decay 0.01) with a linear learning-rate schedule and 10% warm-up. Training was performed for a fixed maximum of 6 epochs, selecting the best checkpoint by validation macro-averaged F1. Batch sizes were 16 for training and 32 for evaluation.

Class imbalance was handled using class-weighted cross-entropy, with class weights computed from the training-set class distribution. Due to computational constraints, each configuration was trained once using a fixed random seed (SEED = 42); uncertainty was quantified using bootstrapped confidence intervals (Section 2.9). All experiments were run on an NVIDIA Tesla T4 GPU with 16 GB of VRAM.

## 3. Results

### 3.1. Dataset Characteristics

After preprocessing and conservative deduplication, the final dataset comprised 394 unique musculoskeletal ultrasonography report segments. The class distribution reflected routine orthopedic practice, with a predominance of benign or indeterminate findings as Table 1.

### 3.2. Overall Performance Comparison

Across all evaluated approaches, Model A (Base BERT) achieved the highest overall test performance, reaching 0.925 accuracy and 0.9106 macro-F1 (Table 3). Keyword fusion and regex post-processing did not improve aggregate metrics; across multiple configurations, they led to reductions in accuracy, macro-F1, or both, primarily by increasing false-positive RED predictions and perturbing otherwise correct baseline decisions.

### 3.3. Internal Performance

Table 2 compares the performance of the BERT model and classical baselines on both patient-level and stratified splits. The BERT model consistently outperforms the baselines in macro-F1 and recall of the RED class. Confidence intervals reflect uncertainty due to the small test set.

Given the limited size of the internal test set (*n* = 40), performance estimates were complemented with 95% confidence intervals derived from bootstrapping. For the patient-level split, the fine-tuned BERT model achieved a macro-F1 score of 0.87 (95% CI: 0.72–0.93), indicating reasonably stable performance despite the small sample size.

The BERT model’s **RED** recall is 80% on the patient-level split. To reduce false negatives we experimented with threshold adjustment but found a trade-off with precision; we recommend clinicians treat the model as an assistive tool and review all reports flagged as non-urgent.

### 3.4. Baseline Cross-Validation and Confidence Intervals

Five-fold group cross-validation of the logistic regression baseline yielded a mean macro-F1 score of 0.86 (95% CI ± 0.02) and a mean accuracy of 0.89 (95% CI ± 0.03), confirming that baseline performance was stable across different patient-level splits. Bootstrapped 95% confidence intervals for the baseline model’s macro-F1 on the internal test set were 0.57–0.93 for the patient-level split and 0.61–0.96 for the stratified split. In comparison, the fine-tuned BERT model achieved bootstrapped macro-F1 confidence intervals of 0.72–0.93 (patient-level split) and 0.75–0.97 (stratified split). We additionally performed a paired bootstrap comparison as an exploratory robustness check. Given the small test set and overlapping confidence intervals, we interpret any observed differences cautiously and do not claim definitive statistical superiority based on a single *p*-value.

### 3.5. Deduplication Sensitivity Analysis

To assess the potential impact of the deduplication strategy on model performance, two sensitivity analyses were conducted focusing on report segments with identical text but conflicting labels. In the first sensitivity analysis, a majority voting strategy was applied to conflicting duplicates, whereby each segment was assigned the label most frequently selected by annotators, and segments without a clear majority were excluded. This approach resulted in a dataset of 385 unique report segments.

Using the same patient-level data splitting and training pipeline, the fine-tuned BERT model achieved an accuracy of 89.2% and a macro-averaged F1 score of 0.85, compared with an accuracy of 90.0% and a macro-F1 score of 0.87 observed with the original deduplication strategy. The differences were small and did not change the overall conclusions.

In a second sensitivity analysis, all report segments with conflicting labels were removed prior to deduplication. This resulted in the same dataset size (*n* = 385) and yielded comparable performance (accuracy = 89.0%, macro-F1 = 0.86), closely mirroring the results of the majority-voting approach.

Overall, these findings demonstrate that alternative deduplication strategies have a minimal impact on classification performance and support the robustness of the proposed approach in handling conflicting duplicate report segments.

### 3.6. Confusion Matrix Analysis

**Figure 1:**
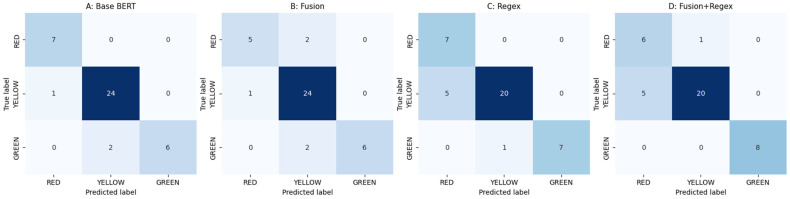
Confusion matrices (GREEN, YELLOW, RED order).

Model A exhibited near-perfect separation of RED in this test split (7/7 correct) and only minor confusion between GREEN and YELLOW (Figure 1). By contrast, Model B reduced recall for RED (5/7 correct) and introduced additional RED ↔ YELLOW confusions. Regex-based models increased the number of YELLOW → RED flips, improving GREEN recall in places but at the expense of precision for RED.

### 3.7. External Document-Level Evaluation on Independent Reports

To assess real-world generalizability, the trained BERT model was evaluated on an independent external dataset consisting of 39 full superficial soft tissue ultrasound reports. Each report was extracted from a distinct radiology report PDF document obtained in 2022. This external dataset was collected independently and was not used during model training, validation, or hyperparameter tuning. Reference labels were assigned by an expert physician blinded to the model outputs. The class distribution of the external dataset was 9 GREEN, 24 YELLOW, and 6 RED reports.

On the external test set, the BERT model achieved an overall accuracy of 82.1% and a macro-averaged F1 score of 0.76. Per-class precision, recall, and F1 scores are summarized in Table 4. Performance was highest for the YELLOW class (precision = 0.79, recall = 0.96, F1 = 0.85), reflecting the predominance of non-urgent but clinically relevant findings. For the RED class, the model achieved a precision of 0.80, recall of 0.67, and F1 score of 0.73, indicating reasonable sensitivity for urgent findings in previously unseen reports. GREEN class performance showed lower recall (0.56), consistent with the subtle linguistic distinction between normal findings and mildly abnormal descriptions.

The corresponding confusion matrix for the external evaluation is presented in Table 5. Error analysis revealed that most misclassifications occurred in borderline YELLOW/RED cases, particularly in reports describing small hematomas versus indeterminate solid masses. These discrepancies primarily reflected clinical ambiguity in reporting language rather than systematic model failure, supporting the use of the proposed system as a decision-support tool in real-world orthopedic workflows.

### 3.8. Error Analysis of Hybrid Heuristics

We examined the impact of keyword fusion and regex-based heuristics on the validation set. A grid search over the fusion weight α (0–3) and gating thresholds showed that the best fusion configuration still underperformed the base BERT model, with consistently lower macro-F1 and RED recall. The regex layer increased the number of RED predictions but at the cost of a higher false-positive rate. Representative report segment examples are provided in Appendix A. Based on these findings, we decided not to include the heuristic components in the final system.

## 4. Discussion

The use of artificial intelligence tools in the medical field is becoming increasingly widespread. Natural language processing (NLP)-based applications, which enable computer-assisted processing of textual data, represent an important component of this field [7,8]. Through NLP-based approaches, it has become possible to automatically analyze large volumes of medical text and extract meaningful information [16]. Textual reports accompanying radiological imaging contain unstructured data and therefore represent a valuable resource for NLP applications. Previous studies investigating the use of NLP for risk analysis and staging, particularly in oncologic imaging reports, have demonstrated promising results [14,15]. In this study, an NLP-based urgency classification system was developed using superficial ultrasonography reports requested for superficial soft tissue masses in an orthopedic outpatient clinic. The successful derivation of an urgency scale from routine clinical ultrasound reports represents a notable and clinically relevant finding, particularly with respect to the early identification of cases requiring rapid clinical response.

In the field of oncology, various studies have demonstrated that NLP can be effective in mass grading, malignancy risk assessment, and staging processes. The literature reports successful outcomes for NLP-based models developed using thyroid ultrasonography reports to evaluate thyroid nodules, breast ultrasonography reports for staging breast lesions, computed tomography reports for the detection of lung nodules, as well as automatic TNM staging of colorectal cancer radiology reports using pre-trained language models [14,15,18,19]. However, a large proportion of existing studies focus on decision-support approaches in which model outputs are presented to clinicians but still require re-evaluation by the clinician. Our study differs from the existing literature in this respect. The developed model aims to identify situations requiring rapid clinical response at an early stage by directly detecting urgency-related expressions within report texts. This approach may help prevent time loss, particularly in suspicious cases that require additional diagnostic tests or further evaluation. As demonstrated in our study, early and effective communication with patients in cases raising suspicion of malignancy and necessitating rapid advanced diagnostic procedures may significantly accelerate diagnostic and treatment processes. This has the potential to positively impact patient survival and clinical outcomes, especially in oncological cases. Furthermore, the model successfully identified non-neoplastic conditions requiring urgent intervention, such as hematomas and abscesses.

Su et al. reported that an NLP-based model developed to identify negative statements in radiology reports demonstrated high sensitivity [20]. Seo et al. described an NLP model for prioritizing appointments in head and neck patients by jointly evaluating imaging and pathology results [21]. The model achieved an accuracy of 81.9% in classifying pathology type and 86.8% in determining urgency level, and demonstrated clinically promising performance in identifying patients requiring urgent care based on malignancy risk. Sugimoto et al. reported high performance in identifying findings suggestive of cancer using chest and abdominal computed tomography reports in a multicenter study [22]. In addition, similar studies have reported successful results for NLP-based triage applications in emergency department settings [16,17]. The performance metrics achieved in our study are comparable to those reported in the existing literature. Notably, despite the use of non-English texts and radiology reports authored by multiple radiologists with varying reporting styles, the careful selection of the training dataset and the stratification of risk-oriented keyword groups contributed to improved model performance. These findings suggest that the methodological framework applied in this study may be transferable to other triage- and urgency-assessment applications based on unstructured clinical text.

By integrating the developed program and similar NLP-based approaches into healthcare information and automation systems, radiology reports may be automatically evaluated after reporting. This could allow both patients and physicians to be promptly informed through automated notifications in urgent situations, thereby preventing potential time delays. However, the content of radiology reports may vary depending on individual radiologists’ styles of expression and terminology preferences, which makes standardization of textual data challenging. In languages other than English, additional variability arising from translation processes and linguistic diversity may further exacerbate this issue. As a result, even high-performing models may be at risk of misclassifying certain reports. Therefore, achieving a degree of standardization in radiology reporting language is essential for the broader adoption of NLP-based systems in clinical practice. Although establishing a fully standardized language for all radiology reports may be difficult in practice, the development of a common urgency or risk-coding framework adopted by radiologists could substantially facilitate the clinical integration of such applications. Furthermore, the ability of NLP-based programs to operate with lower technical requirements compared with image processing-based AI applications may represent an advantage, enabling faster and more widespread integration of these systems into healthcare infrastructures.

A critical safety consideration is the risk of false-negative classification in the RED (urgent) category, which could delay further diagnostic evaluation. In our internal evaluation, RED recall remained high (e.g., 0.80 on the patient-level split), but misclassification cannot be eliminated, especially when reports contain atypical wording or limited descriptive detail. Accordingly, the system should be deployed as a decision-support tool rather than an autonomous triage gate: reports flagged as RED can be prioritized for rapid review, while clinicians should retain responsibility for final urgency decisions. Threshold adjustments intended to further reduce false negatives were explored, but produced an expected trade-off with increased false-positive escalation; this reinforces the need for institution-specific calibration and governance during prospective deployment.

This study has several limitations that should be considered. First, the single-center and retrospective design may limit the generalizability of the findings to other institutions and patient populations. In addition, the analyzed ultrasonography reports were written in a free-text format and exhibited substantial linguistic heterogeneity depending on individual radiologists’ reporting styles and terminology, which may introduce noise affecting the performance of natural language processing models.

Second, report labels were derived from clinical urgency assessments rather than from histopathological confirmation or additional imaging as a gold standard. While this approach reflects real-world triage decision-making processes in orthopedic practice, it may introduce subjectivity into the reference labels. Another relevant limitation is that the dataset consisted exclusively of reports written in Turkish. The limited availability of domain-specific pre-trained NLP models for languages other than English may have influenced model performance and restricted direct transferability to other linguistic settings.

The observed class imbalance in the dataset, particularly the predominance of YELLOW (non-urgent) cases and the underrepresentation of RED (urgent) cases, represents a notable limitation. This distribution reflects routine orthopedic outpatient practice in a non-oncology-focused center, where superficial soft tissue ultrasound examinations are typically requested for benign or indeterminate conditions. Although class imbalance may bias models toward the majority class, we deliberately avoided aggressive resampling or synthetic balancing techniques, as these approaches may distort true clinical prevalence and reduce real-world applicability. Future multicenter studies, particularly those involving oncology-focused practices, may allow for more balanced class distributions and improved assessment of high-risk cases.

Furthermore, external evaluation was performed using reports from the same institution and in the same language as the training data, which limits generalizability to other centers, reporting conventions, and healthcare systems. Radiology reporting practices, particularly in the expression of diagnostic uncertainty, may vary substantially across institutions. While our findings demonstrate the applicability of NLP-based urgency classification in a real-world orthopedic setting, broader generalization will require multicenter validation and adaptation to institution-specific reporting styles.

Finally, this study lacked prospective or user-centered evaluation. The model was assessed retrospectively using existing report data, and its impact on real-time clinical workflows was not directly measured. Future studies should include prospective implementation within clinical information systems, accompanied by user-centered evaluations involving orthopedic surgeons and radiologists to assess usability, alert burden, and clinical acceptance.

## 5. Conclusions

In this study, we developed and evaluated an NLP-based triage system for the automatic classification of superficial soft tissue ultrasound reports into clinically meaningful urgency categories. Using real-world radiology report data, the proposed approach demonstrated high classification performance, particularly in identifying urgent findings that require prompt clinical attention. By directly analyzing unstructured report text, the model provides a practical and scalable solution that complements existing imaging-based artificial intelligence approaches without requiring access to image data.

The ability to automatically flag reports suggestive of malignancy, hematoma, or other conditions requiring rapid intervention highlights the potential clinical utility of this system in routine orthopedic practice. The integration of such NLP-based tools into healthcare information systems may facilitate earlier clinical responses, reduce delays in patient management, and support more efficient triage workflows. Although further multicenter validation and prospective evaluation are warranted, the findings of this study suggest that NLP-driven analysis of radiology reports represents a promising and readily deployable approach for urgency assessment in musculoskeletal imaging.

## Figures and Tables

**Table 1:** Class distribution after preprocessing.

Triage Class	Count	Percentage (%)
GREEN	75	19
YELLOW	248	63
RED	71	18
Total	394	100

This imbalance highlights the clinical relevance of robust discrimination between benign (GREEN), follow-up-requiring (YELLOW), and clinically significant (RED) findings.

**Table 2:** Performance comparison of NLP models on the internal test set, including per-class F1 scores and 95% confidence intervals for macro-F1.

Split Type	Model	Accuracy	Macro-F1 (Mean ± 95% CI)	Per-Class F1 (GREEN/YELLOW/RED)
Patient-level	BERT	0.90	0.87 (0.72–0.93)	0.83/0.93/0.80
	TF-IDF + LR	0.86	0.77 (0.57–0.93)	0.71/0.90/0.65
	SVM	0.86	0.75 (0.55–0.92)	0.69/0.88/0.59
	DistilBERT	0.89	0.85 (0.70–0.91)	0.81/0.91/0.74
	XLM-Roberta	0.88	0.84 (0.68–0.90)	0.79/0.90/0.72
Stratified	BERT	0.93	0.91 (0.75–0.97)	0.90/0.94/0.88
	TF-IDF + LR	0.86	0.84 (0.61–0.96)	0.84/0.92/0.76
	SVM	0.85	0.81 (0.58–0.94)	0.82/0.89/0.71
	DistilBERT	0.90	0.87 (0.72–0.93)	0.86/0.93/0.80
	XLM-Roberta	0.88	0.84 (0.68–0.90)	0.83/0.90/0.75

**Table 3:** Test-set performance (*n* = 40).

Model	Description	Accuracy	Macro-F1	Weighted-F1	Fusion α	Regex Changes
A	Base BERT	0.93 (0.82–1.00)	0.91 (0.75–0.97)	0.92	-	-
B	BERT + keyword fusion	0.86 (0.78–0.95)	0.84	0.87	2.0	-
C	BERT + regex on base	0.85 (0.72–0.95)	0.85	0.86	-	5
D	Fusion + regex	0.85 (0.72–0.95)	0.85	0.86	2.0	7

**Table 4:** Baseline BERT performance on the external test set.

Class	Precision	Recall	F1-Score	Support
GREEN	1.00	0.56	0.71	9
YELLOW	0.79	0.96	0.85	24
RED	0.80	0.67	0.72	6
MacroAvg	0.86	0.73	0.76	-
Accuracy	-	-	0.82	39

**Table 5:** Confusion matrix of baseline BERT predictions on the external test set.

True\Pred	GREEN	YELLOW	RED
GREEN	5	4	0
YELLOW	0	23	1
RED	0	2	4

## Data Availability

The raw data supporting the conclusions of this article will be made available by the authors on request.

## Abbreviations

The following abbreviations are used in this manuscript:

AI	Artificial Intelligence
ML	Machine Learning
NLP	Natural Language Processing
USG	Ultrasonography

## Appendix A

These examples illustrate typical free-text ultrasound report segments used as input for the NLP-based triage model. The examples reflect real-world reporting style and terminology and are provided to enhance transparency and reproducibility of the classification task.

**Table A1:** Representative Report Segments.

*Triage Category*	*Representative Report Segment*
***GREEN***	*“Subcutaneous soft tissues appear normal. No cystic or solid lesion is detected. No pathological finding is identified.”*
***YELLOW***	*“A well-circumscribed cystic lesion measuring approximately 12 mm is observed in the subcutaneous tissue, compatible with a ganglion cyst. Clinical follow-up is recommended.”*
***RED***	*“An ill-defined heterogeneous mass with irregular margins is noted within the soft tissues. Further imaging and urgent clinical correlation are recommended to exclude malignancy.”*
